# D-ß-hydroxybutyrate: an anti-aging ketone body

**DOI:** 10.18632/oncotarget.3423

**Published:** 2015-02-19

**Authors:** Clare Edwards, Neil Copes, Patrick C. Bradshaw

**Affiliations:** Department of Cell Biology, Microbiology and Molecular Biology, University of South Florida, Tampa, FL, USA

During times of starvation or limited carbohydrate consumption when glycogen stores are diminished, triglycerides in adipose tissue are broken down into fatty acids and transported to the liver. There they are catabolized to acetyl-CoA, which is then converted into the ketone bodies acetoacetate, ß-hydroxybutyrate (ßHB), and acetone which are exported from the liver. In other tissues ßHB and acetoacetate are used as energy sources, through their conversion back into acetyl-CoA, which is especially important in neurons where very little fatty acid catabolism takes place. In mitochondria ßHB is broken down into acetoacetate by ßHB dehydrogenase 1 generating NADH. The resulting acetoacetate is metabolized to acetyl-CoA through acetoacetyl-CoA and is further broken down leading to the production of NADH and FADH2 as part of the citric acid cycle. ßHB has shown some effectiveness in the protection against Alzheimer's and Parkinson's disease-mediated neurodegeneration in animal models and human trials [1], but not much is known about its effects on the aging process itself. Moreover, the mechanisms through which ßHB are protective are not entirely clear. Recent evidence suggests that ßHB protects against oxidative stress through its action as an endogenous class I and class IIa histone deacetylase (HDAC) inhibitor to increase expression of oxidative stress resistance factors such as FoxO3A and MT2 (metallothionein 2) [2]. This is not without precedent as the structurally related HDAC inhibitor butyrate has been shown to be protective in mammalian disease models and to extend lifespan in *C. elegans* [3].

ßHB levels have been shown to increase to roughly 1-2 mM levels in the plasma during fasting or to 0.6 mM during calorie restriction (CR) [2], a 20%-40% lowering of caloric intake that consistently extends lifespan and protects against disease [4]. This is in roughly the same concentration range in which ßHB inhibits class I HDACs (IC_50_ =2-5 mM) [2]. Future research aims to identify more of the specific genes whose expression is modified by ßHB and to determine the degree to which CR is protective against aging and disease in mice defective in ketone body production.

We have recently shown that administering ßHB to *C. elegans* nematodes extended lifespan and delayed proteotoxicity and glucose toxicity [5]. D-ßHB, but not L-ßHB, extended C. elegans lifespan in a sirtuin SIR-2.1 and AMP kinase-dependent manner that also required the stress-responsive transcription factors DAF-16, an ortholog of mammalian FOXO genes and SKN-1, an ortholog of mammalian Nrf genes including Nrf2 (NFE2L2). Since ßHB did not extend lifespan under dietary restriction, ßHB likely acts as a dietary restriction mimetic, as previously hypothesized [6]. Even though the protective effects of ßHB have already been shown in rodent disease models, this was the first report to identify ßHB as a positive modulator of lifespan in wild-type animals. ßHB extended the lifespan of *C. elegans*, but this lifespan extension was almost completely eliminated in worms where either the histone deacetylase (HDAC) *hda-2* or the HDAC *hda-3* was knocked down. In addition knockdown of these HDACs in the absence of ßHB addition extended lifespan supporting a role for ßHB-mediated HDAC inhibition in lifespan extension [5].

**Figure 1:**
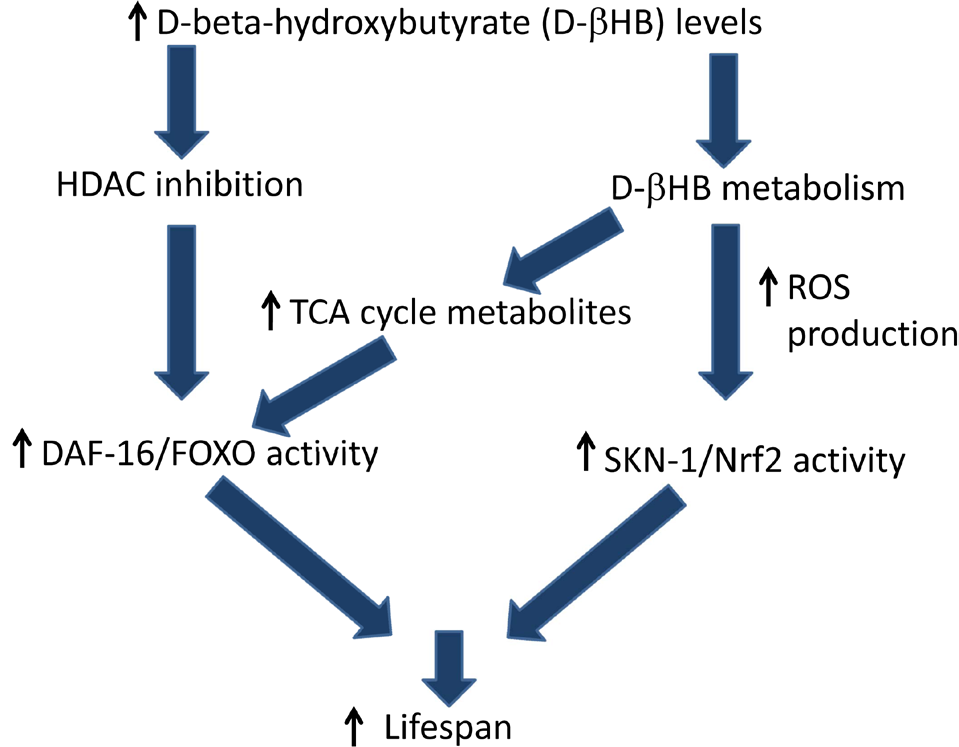
Proposed mechanisms of D-ßHB mediated lifespan extension in *C. elegans*.

We propose two overlapping pathways for lifespan extension mediated by ßHB supplementation as shown in Figure 1. In the first, ßHB directly inhibits HDACs to increase histone acetylation leading to DAF-16/FOXO transcription and activation. The second proposed protective pathway involves mitochondrial metabolism of ßHB, leading to increased citric acid cycle metabolism and electron transport chain (ETC) activity, increased reactive oxygen species (ROS) production, and activation of the SKN-1 antioxidant response pathway. We observed decreased ßHB-mediated lifespan extension in a mitochondrial complex I-defective mutant supporting this mechanism. Interestingly, the SKN-1 target gene F55E10.6, a short-chain dehydrogenase/reductase with ßHB dehydrogenase activity was also required for ßHB-mediated lifespan extension. In addition SKN-1 activation has been shown to repress expression of the insulin-like peptides DAF-28 and INS-39, decreasing DAF-2 insulin receptor signaling to activate DAF-16 [7]. Several unpublished pieces of data from our lab support the model of ßHB-mediated lifespan extension proposed in Figure 1 including the strong reduction of lifespan extension in *daf-16* mutant worms compared to wild-type worms when both were fed *hda-2* or *hda-3* RNAi clones, and the failure of *hda-2* or *hda-3* RNAi clones to activate a SKN-1 transcriptional reporter strain.

Lastly, we showed that ßHB increased thermotolerance and delayed amyloid beta-induced paralysis and decreased alpha-synuclein aggregation in *C. elegans* models of Alzheimer's and Parkinson's diseases. Although compelling evidence for the use of ßHB as a prophylactic for disease in nematode models was shown, questions remain. For example, are the signaling pathways mediating ßHB-mediated lifespn extension also required for protection against amyloid-beta and alpha-synuclein toxicity? Future experiments will further elucidate the molecular mechanisms responsible for the protective effects of ßHB and this knowledge will allow for a broader use of ßHB as a therapy for aging-related disorders.
